# Primary Lateral Sclerosis: Implications for Diagnostic Criteria From a Natural History Study in the Netherlands

**DOI:** 10.1212/WNL.0000000000213461

**Published:** 2025-05-19

**Authors:** Bálint S. de Vries, Eva Maria Johanna de Boer, Frans Brugman, Philip Van Damme, Jan Herman Veldink, Michael A. van Es, Leonard H. van den Berg

**Affiliations:** 1 Department of Neurology, Brain Center Rudolf Magnus, University Medical Center Utrecht, the Netherlands;; 2 ACIBADEM International Medical Center, Amsterdam, the Netherlands;; 3 Department of Neurology, University Hospitals Leuven, Belgium; and; 4 Laboratory of Neurobiology, Department of Neuroscience, KU Leuven and Leuven Brain Institute, KU Leuven, Belgium.

## Abstract

**Background and Objectives:**

Primary lateral sclerosis (PLS) is a rare disease characterized by upper motor neuron (UMN) degeneration. We aimed to elucidate the natural history in patients with UMN syndromes suggestive of PLS and validate the most recent diagnostic (consensus) criteria.

**Methods:**

A validation study of a long-term follow-up cohort was conducted, including adults with UMN syndromes and disease durations ≥6 months. Patients were assessed at baseline (T1), at 3 years (T2), and when possible after 13 years (T3). Diagnostic categorization followed the 2020 PLS consensus criteria. Main outcomes included diagnostic classification at follow-up and survival.

**Results:**

The study comprised 86 patients (34 women [40%], mean age 58.9 ± 10.1 years), of whom 43 met the PLS consensus criteria at baseline (6 probable, 37 definite). Eight patients had a disease duration <2 years, and 35 patients presented with UMN symptoms localized to 1 region (1 bulbar, 34 legs). Change of initial diagnosis occurred in 14% of patients with PLS, and 49% of patients presenting with UMN symptoms in 1 region progressed to PLS. Seven patients developed amyotrophic lateral sclerosis (ALS), and for 7 patients, diagnosis was revised to hereditary spastic paraplegia (HSP). Survival was shorter for patients with a disease duration <4 years. In the probable PLS group, 33% converted to ALS. Converters had a steeper Amyotrophic Lateral Sclerosis Functional Rating Scale slope (*p* = 0.023) and shorter symptom duration (*p* < 0.001) at inclusion. Of patients presenting with leg symptoms, diagnosis was revised between T2 and T3 in 29%. Introducing a 4-year minimal disease duration for PLS diagnosis and categorization based on regions involved resulted in 86% of PLS diagnoses remaining within the PLS category, 5% transitioning to ALS (slow variant), and 9% to HSP. Survival was longest for patients presenting with symptoms confined to arms and legs or legs only, followed by those with bulbar involvement at baseline, while patients with disease durations between 6 months and 4 years exhibited the shortest survival.

**Discussion:**

Our findings suggest that a diagnosis of PLS should be deferred until 4 years after symptom onset because shorter durations correlate with higher ALS conversion rates and shorter survival. Categorization by regional involvement may facilitate more effective monitoring of patients with UMN syndromes.

## Introduction

Primary lateral sclerosis (PLS) is a rare, late-onset neurodegenerative disorder primarily affecting upper motor neurons (UMNs), leading to spasticity, mobility loss, and mild weakness. Although PLS is a disabling disorder, it is not necessarily life-shortening, with survival often exceeding 15 years.^1^ The most common patterns of onset and progression are bulbar onset with later progression to the arms and legs, relatively symmetrical onset in the legs with progression to the arms and bulbar region, or markedly one-sided onset and progression, also known as Mills syndrome.

PLS is diagnosed by exclusion and, therefore, challenging because the initial differential diagnosis is broad, including conditions such as metabolic myelopathies (adrenomyeloneuropathy), primary progressive multiple sclerosis, Alexander disease, autoimmune diseases (anti-amphiphysin antibodies), infections (human T-lymphotropic virus types 1 and 2, syphilis), and paraneoplastic syndromes (e.g., in patients with breast cancer).^2,3^ Most conditions in this differential diagnosis can be demonstrated through imaging and genetic and laboratory testing. In patients where ancillary investigations turn out negative, a relatively short, but phenotypically overlapping, list of diagnostic considerations remains, mainly consisting of neurodegenerative diseases: PLS, UMN-predominant presentations of amyotrophic lateral sclerosis (ALS), (sporadic) hereditary spastic paraplegia (HSP), and PLS-progressive supranuclear palsy.

In many patients, the diagnosis becomes clear over time as additional signs and symptoms develop. For instance, signs of lower motor neuron involvement may become apparent, indicating that the diagnosis is ALS rather than PLS. Therefore, the various diagnostic criteria for PLS have emphasized a minimum symptom disease duration (varying from 2 to 4 years), in which the clinical phenotype should remain a pure UMN disorder.^2,4,5^ For patients, this unfortunately means that there is an extended period of diagnostic uncertainty.

The latest diagnostic criteria for PLS, published in 2020, require progressive UMN involvement for 2 years in at least 2 of 3 regions (bulbar, upper extremity, lower extremity), a minimum age of 25 years at diagnosis, and the absence of both sensory symptoms and lower motor neuron degeneration, and that alternative diagnoses have been ruled out. There are 2 categories: “probable PLS (P-PLS),” with a disease duration of 2–4 years, and “definite PLS (D-PLS),” with a disease duration of 4 years or longer. Patients with progressive UMN involvement in 1 region are not considered to have PLS. The criteria do not provide guidance on how long patients with UMN involvement in 1 region should be followed or when PLS can be dismissed as diagnosis.^2^

The current criteria are based on international consensus but have not been validated extensively. In this study, we present long-term follow-up data from a cohort of patients in whom PLS was considered to be the most likely diagnosis. We subsequently applied the 2020 PLS diagnostic criteria to this cohort with the primary objective of validating the diagnostic accuracy of the 2020 consensus criteria.

## Methods

### Patient Inclusion and Follow-Up

The study population was recruited between November 2002 and March 2005. During this period, all Dutch neurologists and 1 large referral center for MND in Belgium (Leuven) were requested to enroll patients suspected of having PLS in this study.

The inclusion criteria for our study were as follows: (1) a gradually progressive UMN syndrome, (2) age at onset 18 years or older, (3) disease duration of at least 6 months, and (4) no evidence for an alternative diagnosis based on ancillary investigations.

The exclusion criteria were as follows: (1) positive family history for ALS or HSP at inclusion, (2) sensory signs, (3) cerebellar or extrapyramidal signs, or (4) LMN involvement at inclusion. Regarding LMN involvement, we accepted patients with mild focal amyotrophy of the interosseous muscles of the hands, or of the anterior tibial or calf muscles, or fasciculations in muscles without weakness or atrophy. Patients were excluded if the EMG criteria for ALS were met at inclusion (evidence of both chronic neurogenic change and acute denervation, as defined in the revised El Escorial criteria [rEEC]).^6^

Medical records were reviewed, and all patients were evaluated by F.B. and L.v.d.B. (visit T1). Patients were followed at 9-month intervals for a total of 3 years after enrollment (visit T2 was at 3 years of follow-up), hereby ensuring that all participants had a minimum symptom duration >3 years, as required by the relevant diagnostic criteria for PLS at that time.^3^ Visit T3 took place 10 years after visit T2 and was performed by B.d.V. Patients who were unwilling or unable to participate in the clinical follow-up were contacted by telephone.

At each visit, a history was taken and a neurologic examination was performed to determine which regions (as defined in the rEEC) were affected.^6^ The bulbar region was considered affected if pseudobulbar dysarthria and/or dysphagia was present. The arms and legs were considered to be affected if there was at least one of the following findings on neurologic examination: spasticity (defined as a modified Ashworth Scale score ≥2),^7^ obvious loss of dexterity due to spasticity, pathologic hyperreflexia (defined as Institute of Neurological Diseases and Stroke myotatic reflex scale score 4 or 5 or extensor plantar response),^8^ or UMN related paresis ≥Medical Research Council grade 4.^9^

The degree of disability was measured using the Amyotrophic Lateral Sclerosis Functional Rating Scale (ALSFRS) or ALSFRS-revised (ALSFRS-R) from 2007 onward.^10,11^ The rate of functional decline was calculated by the number of points lost on the ALSFRS or ALSFRS-R divided by disease duration in years (assuming a maximum score at onset).

Survival status of patients was obtained on August 31, 2023.

For patients who were deceased, the date of death and (when available) cause of death were obtained. Causes of death were subsequently categorized as either related, possibly related, unrelated to PLS, or unknown. We considered severe disability with secondary nutritional problems, pulmonary infections due to aspiration in patients with bulbar symptoms, and euthanasia as PLS-related. Cause of death was regarded as possibly PLS-related if patients died from conditions that could be viewed as complications of PLS, such as infections or falls. If cause of death was clearly attributable to a different condition (for example, cancer), deaths were categorized as unrelated to PLS. Finally, for some patients, we were unable to retrieve the cause of death, resulting in the category “unknown.” All causes of death were independently categorized by B.d.V. and E.d.B. Discrepancies were discussed and labeled according to consensus.

### Standard Protocol Approvals, Registrations, and Patient Consents

All patients provided informed consent to participate. This study was conducted in accordance with the 1964 Helsinki Declaration and approved by the medical ethics review board of the University Medical Center Utrecht, and written informed consent was obtained from all participants. The STROBE checklist was used while writing the article.

### Classification of Patients

Retrospective application of the current consensus criteria at visit T1 resulted in 5 groups of patients. Patients who fulfilled the criteria for either P-PLS (disease duration 2–4 years) or D-PLS (disease duration ≥4 years)^2^ and patients who did not meet the current criteria were categorized as “undetermined” (those with a disease duration between 6 months and 2 years), “bulbar only” (restricted UMN bulbar symptoms with a disease duration ≥2 years), and “lower limbs only” (UMN symptoms restricted to the lower limbs with a disease duration ≥2 years), which is a phenotype most accurately described as an insidiously progressive spastic paraplegia.

### Genetic Testing

At the time of enrollment in the study, the availability and extent of genetic testing was limited. At inclusion, all patients were screened for the HSP genes considered to be most relevant at that time (*Spastin* [*SPG4*], *Paraplegin* [*SPG7*], and *BSCL2* [*SPG17*]). Patients with UMN signs in the bulbar region or the arms were also screened for variants in *Alsin* (*ALS2*). Patients were excluded if a pathogenetic variant was identified at baseline.

Over the past years, considerable progress has been made in our understanding of the genetics of ALS and HSP. All DNA samples of patients without bulbar symptoms were, therefore, tested again in 2018 for variants in an updated panel of HSP genes (n = 48) using next-generation sequencing (eTable 1).

### Statistical Analyses

Differences in clinical characteristics between groups at inclusion were tested using the Pearson χ^2^ test, independent-samples *T* test, Mann-Whitney *U* test, and analysis of variance (Table 1). The ALSFRS was used to estimate the rate of decline of functional status during follow-up. In our study, the ALSFRS was initially applied and later replaced by the ALSFRS-R. To permit analyses of both the ALSFRS and ALSFRS-R, we omitted the 2 items only present in the ALSFRS-R: orthopnea and the need for ventilatory support. To estimate the rate of decline of functional status during follow-up (ALSFRS), linear mixed-effects models were used. Kaplan-Meier methods and log-rank tests were used to study survival data.

**Table 1:** Clinical Characteristics at Baseline of 86 Patients With a Sporadic Adult-Onset Upper Motor Neuron Syndrome, Classified According to the Turner Consensus Criteria

	Definite PLS (≥4 y, ≥2 affected regions) (n = 37)	Probable PLS (2–4 y, ≥2 affected regions) (n = 6)	Lower limbs only (≥2 y, legs only) (n = 34)	Bulbar region only (≥2 y, bulbar only) (n = 1)	Undetermined (6 mo–2 y) (n = 8)	*p* Value
Male, n (%)	23 (62)	1 (17)	21 (62)	1 (100)	6 (75)	0.187
Disease duration, y, mean (range)	10 (4–25)	3.2 (2.3–4)	9 (2.3–31)	3	1.2 (0.5–1.9)	<0.001
Age at onset, y, mean (range)	50 (32–68)	60 (48–76)	47 (18–63)	62	59 (45–74)	0.003
Onset site, n (%)						
Bulbar	4 (11)	1 (17)	0	1 (100)	3 (38)	
Arms	0	2 (33)	0	0	2 (25)	
Legs	33 (89)	3 (50)	34 (100)	0	3 (38)	
Affected body regions at inclusion, n (%)						
Bulbar	26 (70)	3 (50)	0	1 (100)	6 (75)	
Arms	33 (89)	4 (67)	0	0	4 (50)	
Legs	36 (97)	6 (100)	34 (100)	0	7 (88)	
EMG findings, n (%)						
Normal	12 (32)	2 (33)	15 (44)	1 (100)	3 (38)	0.619
Minor abnormalities^a^	17 (46)	4 (67)	14 (41)	0	5 (62)	
Missing	8 (22)	0	5 (15)	0	0	
ALSFRS score, mean (range)	26 (12–37)	31 (25–36)	33 (15–39)	36	31 (17–38)	<0.001
ALSFRS slope, mean (range)	−1.6 (0.5–3.5)	−3.2 (1.1–5.5)	−0.9 (0.1–3.4)	−1.17	−6.7 (1.7–9.7)	<0.001

Abbreviations: ALSFRS = Amyotrophic Lateral Sclerosis Functional Rating Scale (maximum 40), ALSFRS slope was calculated as the average yearly change from onset (40 points) to inclusion; LMN = lower motor neuron; mild LMN signs on EMG = needle EMG abnormalities not fulfilling revised El Escorial criteria for probable laboratory-supported ALS; PLS = primary lateral sclerosis.

a EMG finding suggestive of LMN degeneration/reinnervation but not fulfilling the El Escorial or Awaji criteria for ALS.^6,14^

Several patients went on to develop ALS. We compared patient characteristics between patients who developed ALS and those who did not. ALSFRS slope, disease duration at baseline, and age at onset were compared using independent *T* tests, and sex, region of onset, bulbar involvement, and EMG abnormalities (at inclusion) were compared by the χ^2^ test.

Statistical analysis was performed using SPSS statistical software version 29 (SPSS, Chicago, IL) and R (version 4.3.0).

### Declaration of AI Technologies

During the preparation of this work, the authors used ChatGPT from OpenAI to improve language and readability of parts of the text. After using this tool, the authors reviewed and edited the content as needed and take full responsibility for the content of the publication.

### Data Availability

Data not published within this article may be shared (anonymized) at the request of any qualified investigator.

## Results

### Study Population and Follow-Up

A total of 86 patients were included in the study. The baseline characteristics are presented in Table 1. At inclusion, 43 patients met the current consensus criteria, 6 with P-PLS and 37 with D-PLS. Eight patients had a disease duration shorter than 2 years (undetermined group). The remaining 35 patients did not meet the 2020 consensus criteria because UMN symptoms were limited to 1 region. These patients were classified as lower limbs only (spastic paraplegia) or bulbar region only. A total of 4 patients had symptom onset in the arms (n = 2 in the P-PLS group and n = 2 in the undetermined group); however, an “upper limbs only” subgroup was not established because these patients exhibited UMN symptoms in multiple regions at the time of inclusion.

We found significant differences between the different groups at baseline for age at onset (Table 1). Patients with P-PLS were significantly older, whereas the lower limbs only (spastic paraplegia) group was younger. The disease duration was shorter in the P-PLS, undetermined, and bulbar region only groups compared with the D-PLS and lower limbs only groups. The ALSFRS score was lowest in the D-PLS group. ALSFRS slope was significantly highest (more rapid decline) in the P-PLS and undetermined groups. All patients were seen at baseline (T1) and at 3 years (T2), and 28 patients were also seen approximately 10 years after T2, therefore having a 13-year follow-up (T3).

During follow-up, the diagnosis was revised to ALS for patients who developed progressive and generalized LMN loss and to HSP for patients who had a genetic variant associated with HSP revealed by updated genetic testing during the study. Finally, 16 patients in the lower limbs only (spastic paraplegia) group developed UMN involvement in additional regions and, therefore, received the diagnosis PLS.

**Figure 1:**
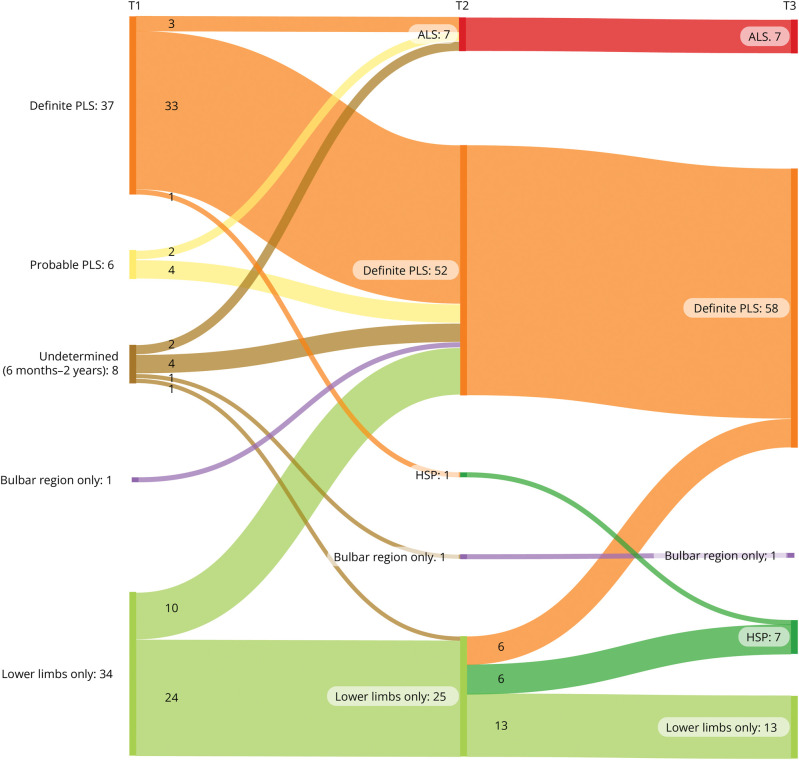
Diagnosis at Each Time Point Based on the 2020 Consensus Criteria for PLS The numbers represent the number of patients per category at each time point. T1 = baseline; T2 = 3 years of follow-up; T3 = approximately 13 years of follow-up. ALS = amyotrophic lateral sclerosis; HSP = hereditary spastic paraplegia; PLS = primary lateral sclerosis.

The Sankey plot in Figure 1 displays the diagnosis at each of the 3 time points according to the 2020 consensus criteria. T1 shows the categories at baseline, as provided in Table 1.

The clinical characteristics of patients who developed ALS or were diagnosed with HSP at later time points are provided in Table 2.

**Table 2:** Characteristics of Patients Who Fulfilled (Revised) El Escorial Criteria for Clinically Probable or Probable Laboratory-Supported ALS During Follow-Up or Who Received the Diagnosis HSP

Case	2020 PLS consensus criteria categorization	Regional categorization	Site of onset	Decade of onset	DD at inclusion	DD at ALS diagnosis	Total DD	EMG abnormalities	El Escorial criteria^13^	ALSFRS slope at inclusion (points lost/year)
1	Undetermined	Undetermined	Bulbar	50	0.5	0.8	2.2^a^	No	Clinically Prob.	−9.7
2	Undetermined	Undetermined	Legs	50	0.7	1.1	3.6^a^	No	Clinically Prob.	−9.5
3	Probable PLS	Undetermined	Legs	60	2.3	2.5	2.7^a^	Yes	Clinically Prob.	−5.5
4	Probable PLS	Undetermined	Arms	60	2.8	3.7	5.9^a^	Yes	Prob. – Lab. Supp.	−4.2
5	Definite PLS	PLS upper and lower limbs	Legs	40	5.5	9.4	16.8^b^	Yes	Prob. – Lab. Supp.	−1.8
6	Definite PLS	PLS with bulbar involvement	Bulbar	60	5.1	6.7	12.9^a^	Yes	Prob. – Lab. Supp.	−3.1
7	Definite PLS	PLS with bulbar involvement	Legs	40	6.7	8.8	22.8^a^	Yes	Prob. – Lab. Supp.	−3.0

Case	2020 PLS consensus criteria categorization	Regional categorization	Site of onset	Decade of onset	DD at inclusion	DD at HSP diagnosis	Total DD	EMG abnormalities	Reason for HSP diagnosis	ALSFRS slope at inclusion (points lost/year)
8	NA (1 region)	PLS lower limbs	Legs	50	12	missing	29^a^	Yes	Family members with spastic paraparesis	−0.2
9	NA (1 region)	PLS lower limbs	Legs	20	30	43	52^b^	Yes	*SPAST* variant (SPG4)	−0.2
10	NA (1 region)	PLS lower limbs	Legs	40	8	22	28^b^	No	*HSPD1* variant (SPG13)	−0.4
11	NA (1 region)	PLS lower limbs	Legs	30	14	29	19^a^	Yes	*ALT1* variant (SPG3A)	−0.2
12	NA (1 region)	PLS lower limbs	Legs	40	5	17	24^b^	Missing	*CYP7B1* variant (SPG5A)	−0.4
13	NA (1 region)	PLS lower limbs	Legs	30	3	17	22^b^	Missing	*SPG7* variant (SPG7)	−1.6
14	Definite PLS	PLS upper and lower limbs	Legs	30	6	7	26^b^	No	Family members with spastic paraparesis	−0.6

Abbreviations: ALS = amyotrophic lateral sclerosis; ALSFRS = Amyotrophic Lateral Sclerosis Functional Rating Scale; Clinically Prob. = clinically probable ALS; DD = disease duration; HSP = hereditary spastic paraplegia; Prob. – Lab. Supp. = laboratory-supported ALS; NA = not applicable; PLS = primary lateral sclerosis.

Data are numbers of years, unless otherwise stated. Categorization PLS is at study inclusion. Total disease duration = onset to death or onset to last verification 31-8-2023. ALSFRS slope was calculated as the average yearly change from onset (40 points) to inclusion.

a Death.

b Alive at last verification.

Overall, based on the longitudinal data, 6 patients (14%) who met the consensus criteria for PLS received an alternative diagnosis after 3 years of follow-up: 5 to ALS and 1 to HSP. Of the group of patients with UMN involvement restricted to 1 region, 17 (49%) progressed to D-PLS (11 patients at T2 and 6 patients at T3).

### Progression to ALS

Of the 37 patients with D-PLS, 3 patients (8%) developed ALS. All these cases had limited atrophy of intrinsic hand muscles at inclusion but did not have more widespread LMN loss on clinical examination or EMG. Repeated needle-EMG studies, however, revealed findings fulfilling laboratory-supported ALS according to the rEEC (Table 2) at a disease duration of 6.7, 8.8, and 9.4 years, respectively.

Of the 6 patients with P-PLS, 2 (33%) developed ALS (cases 3 and 4) at a disease duration of 2.5 and 3.7 years, respectively. In case 3, there were clear clinical LMN signs with profound atrophy and weakness in the arms and legs. In case 4, follow-up needle-EMG studies showed findings in cervical and thoracic regions fulfilling the EEC.

In total, 5 of 43 patients with PLS (D-PLS or P-PLS) developed ALS (11.6%). Patients with PLS who developed ALS had a steeper ALSFRS slope (*p* = 0.023) and shorter symptom duration (*p* < 0.001) at inclusion. Age at symptom onset, sex, region of symptom onset, bulbar involvement at inclusion, and EMG abnormalities at inclusion did not differ significantly. *C9orf72* repeat expansions were tested for 32 of the 86 patients; all were found to be wild type.

### HSP

In 34 patients, UMN involvement was restricted to the lower limbs at baseline. The updated DNA diagnostics yielded pathogenic variants in HSP genes in 5 of these patients (Table 2). Pathogenic variants were identified in *SPAST*, *HSPD1*, *SPG3A*, *CYP7B1*, and *SPG7*. The variants in *SPAST* and *SPG7* were not detected at inclusion but during the follow-up of this study because of improved sequencing methodology with higher coverage. The initial family histories for cases 8 and 14 were negative. However, during follow-up, it became apparent that both had multiple family members developing a slowly progressive, young-onset UMN syndrome, predominantly affecting the lower limbs. Therefore, cases 8 and 14 were also considered to have HSP, although no pathogenic variant was identified through DNA testing. In 13 patients, UMN signs remained restricted to the lower limbs after 13 years of follow-up, which would suggest that the most likely diagnosis is DNA-negative, sporadic HSP.

### Progression to D-PLS

During follow-up, patients from all groups progressed to D-PLS. Four of the 6 patients with P-PLS (66%) progressed to D-PLS (all at T2), as well as 4 of the 8 patients in the undetermined group (all at T2). At T1, 1 patient had bulbar symptoms only with a disease duration over 2 years. Over time, UMN signs became generalized with progression to D-PLS at T2.

A total of 16 patients with UMN involvement restricted to the lower limbs (spastic paraplegia) developed UMN signs in additional regions, thus a phenotype compatible with D-PLS (10 patients at T2 and 6 patients at T3).

### Undetermined UMN Group

Of the 8 patients in the undetermined group, 2 patients were diagnosed with ALS and 4 with D-PLS. In 1 patient, UMN involvement remained isolated to the bulbar region at T2 and T3, and in the remaining patient, UMN signs remained restricted to the lower limbs.

## Discussion

In this study, we longitudinally followed 86 patients presenting with an UMN syndrome with the aim to provide insight into the natural history of this specific patient group and to validate the current consensus criteria for PLS in our cohort. At inclusion, 50% met the criteria for PLS, 41% had only UMN symptoms in 1 region, and 9% had a disease duration between 6 months and 2 years. When following the patients over time and applying the current consensus criteria, 14% of the patients who initially met the criteria for PLS ended up with another diagnosis (ALS or HSP). When looking at the newly added category of P-PLS specifically, 33% converted to ALS within 3 years of follow-up.

The aims of the 2020 consensus criteria for PLS were to reduce diagnostic delay, optimize therapeutic trial design, and catalyze the development of disease-modifying therapy.^2^ To achieve these objectives, it is crucial to demonstrate that these criteria indeed yield high diagnostic accuracy and, therefore, that validation studies are performed.

Thus far, only 1 validation study has been undertaken in a German cohort of patients, diagnosed with PLS based on expert opinion, and to which the current consensus criteria were applied retrospectively.^12^ This study reported diagnosis at baseline and at the last follow-up with a median disease duration of 6.1 years (interquartile range 4.1–12.1) for 41 patients. There were 3 categories: those not meeting the consensus criteria (n = 22), those with P-PLS (n = 9), and those with D-PLS (n = 10).

Of the 22 patients in the “consensus criteria not met” group, 4 developed ALS and 1 was found to have nonprogressive spastic monoparesis of the left leg, later attributed to myelopathy associated with spinal epidural lipomatosis (21 years after symptom onset). Nine patients did go on to develop PLS, and 8 patients still had a clinically pure UMN phenotype that did not meet the criteria.

The diagnosis was not revised for any of the 19 patients meeting the criteria for P-PLS or D-PLS. Hence, the authors conclude that the consensus criteria revealed high diagnostic certainty and prognostic significance.

Although the German validation study found high diagnostic certainty, it is important to note that the population size was modest and that previous studies have found that patients who would fulfill P-PLS or D-PLS criteria developed ALS at later time points. For instance, Gordon et al.^5^ found that 13 patients with PLS from a cohort of 29 (45%) received the diagnosis ALS (77% before 4 years and 23% after 4 years), and Paganoni et al.^13^ found that 2 of 250 patients (0.8%) who fulfilled the Pringle criteria were diagnosed with ALS during follow-up in a large retrospective registry (NEALS). In our study, we found that 8% of patients with D-PLS and 33% of those with P-PLS went on to develop ALS.

Considering that patients with both P-PLS and D-PLS went on to develop ALS and patients from the category lower limbs only progressed to PLS, we explored whether adaptations to the diagnostic criteria would lead to higher diagnostic accuracy and more prognostic value by implementing the following adjustments:

1. Classifying patients according to involved regions that would allow the categories to more accurately reflect the phenotypes, including survival.
2. Extending the minimum duration of UMN involvement to 4 years, instead of 2 years, as a requirement for diagnosing PLS, with the objective of minimizing the number of patients converting to ALS.

Applying these adjustments results in 4 different groups: (1) “PLS bulbar involvement” for patients with UMN symptoms of the bulbar region (other regions may also be involved); (2) “PLS upper and lower limbs”; (3) “spastic paraplegia” (for those with UMN involvement limited to the lower limbs); and (4) “UMN disease (UMND)” a term we propose for patients with a progressive UMN syndrome and a disease duration <4 years.

**Figure 2:**
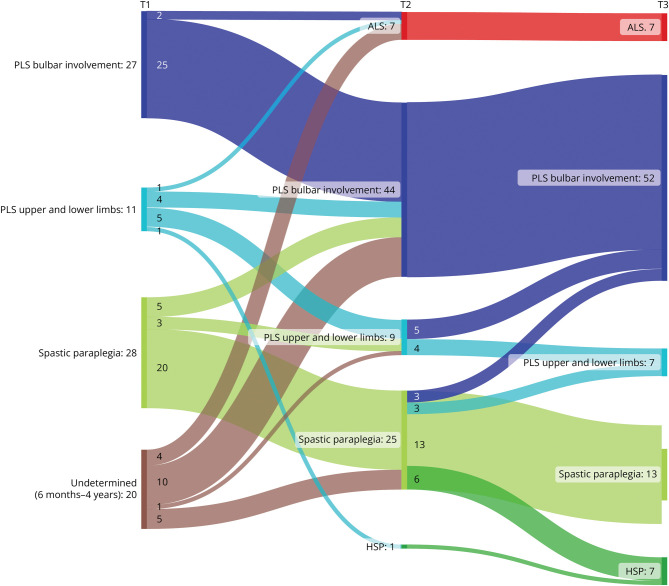
Diagnosis at Each Time Point Based on the Adapted Criteria The numbers represent the number of patients per category at each time point. T1 = baseline; T2 = 3 years of follow-up; T3 = approximately 13 years of follow-up. ALS = amyotrophic lateral sclerosis; HSP = hereditary spastic paraplegia; PLS = primary lateral sclerosis.

Application of the adapted criteria would initially classify a lower percentage of the cohort as PLS (44% vs 50%). However, this would result in similar numbers after 3 years of follow-up (±68%) and, importantly, a lower conversion rate to ALS (8% vs 12%) (Figure 2). It is important to note that in the patients who converted to ALS under the adapted criteria, the diagnosis was based on needle-EMG findings, but not on clinically evident LMN signs. Moreover, all these patients had long survival (13–23 years since onset).

**Figure 3:**
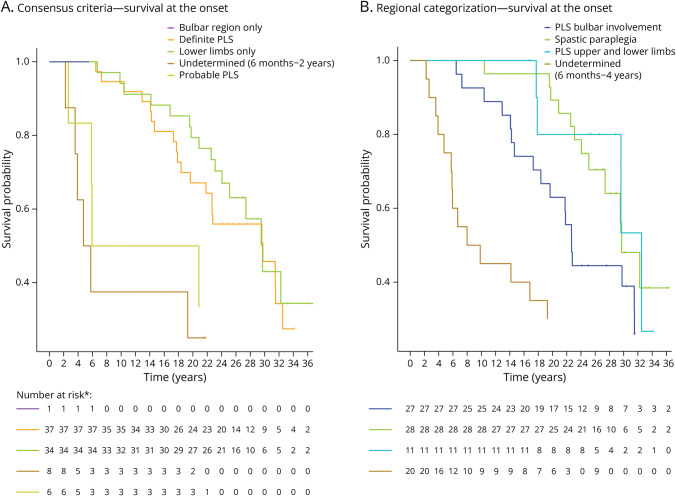
Survival Curves According to 2020 PLS Consensus Criteria and Regional Categorization (A) Kaplan-Meier curves for the different diagnostic groups included in this study according to the current consensus criteria. There is a statistically significant shorter survival for the P-PLS and undetermined groups compared with the D-PLS and lower limbs only groups. There is no difference between P-PLS and the undetermined groups or between the D-PLS and lower limbs only groups. Because there was only 1 patient with bulbar involvement restricted to the bulbar region, this case was not included in the figure. (B) Kaplan-Meier curves for the different diagnostic groups when categorized according to the adapted regional criteria. This results in 3 distinct survival curves for the undetermined group, PLS with bulbar involvement group, and spastic paraplegia group. The PLS with lower and upper limb involvement group shows a similarly long survival distinct from the undetermined group. *The tables with number at risk are affected by missing follow-up data. D-PLS = definite PLS; PLS = primary lateral sclerosis; P-PLS = probable PLS.

We also compared the survival curves for patients based on the current criteria with the curves when applying our proposed adaptations. Application of the current criteria shows that survival is significantly shorter for the undetermined and P-PLS groups compared with the D-PLS and the lower limbs only groups. It seems this shorter survival in the undetermined and P-PLS groups can be attributed to a higher rate of conversion to ALS (Figure 3A). With the proposed adaptations, there are 3 clearly distinct survival curves for the UMND, PLS with bulbar involvement, and spastic paraplegia groups. The PLS upper and lower limbs group is small, but the survival curve is also clearly distinct from the UMND group (Figure 3B).

All studies on PLS have the obvious limitation that they are relatively small, which is inherent given that PLS is a rare disorder. As a result, the estimates of conversion rates to ALS have been variable and are likely inaccurate. Therefore, larger studies on conversion rates to ALS are clearly warranted, as well as more detailed analysis of which factors are associated or predictive of conversion. In this study, ALSFRS decline and symptom duration were the most important factors. However, additional factors, such as EMG changes and serum/CSF neurofilaments, should also be interrogated in future studies.

Both our study and the German validation study enrolled patients who initially did not meet the criteria but did do so during follow-up.^12^ The current criteria do not provide guidance on how long patients should be followed. This could potentially cause the diagnosis of PLS to be missed in patients in whom spread of UMN signs to additional body regions is slow. Conversely, there are patients with predominant UMN signs that remain restricted to the lower limbs for well over a decade and who do not meet PLS criteria. They have negative family histories and DNA testing for HSP and do not fall into a clear diagnostic category. These cases are perhaps also best labeled according to their phenotype, spastic paraplegia.

In summary, this study provides long-term follow-up data (>10 years) on a large cohort of patients with PLS. Using these data, we performed a validation study of the current consensus criteria, which demonstrates several key points for potential improvement and where additional research is needed. First, extending the minimally required duration of a pure UMN phenotype to 4 years would lower the rate of patients with PLS converting to ALS. Given the limited number of patients in our cohort with a disease duration of 2–4 years, our study does not specifically address the optimal time cutoff for diagnosing PLS.

Larger studies on the frequency and timing of the emergence of LMN signs in patients with pure UMN syndrome are required. A better understanding of which factors are predictors of conversion (such as ALSFRS slope) could potentially also be integrated into the updated criteria. This study also demonstrates that long-term follow-up of patients is crucial because patients who initially do not meet PLS criteria may do so at later time points. In our view, updated criteria should contain guidance on how follow-up should be structured. Finally, we propose that classifying patients according to involved body regions, rather than only on disease duration, holds more prognostic value.

## Glossary

ALS: amyotrophic lateral sclerosis
ALSFRS: Amyotrophic Lateral Sclerosis Functional Rating Scale
ALSFRS-R: ALSFRS-revised
D-PLS: definite PLS
HSP: hereditary spastic paraplegia
PLS: primary lateral sclerosis
P-PLS: probable PLS
rEEC: revised El Escorial criteria
UMN: upper motor neuron
UMND: UMN disease
